# The Role of Peptide Signals Hidden in the Structure of Functional Proteins in Plant Immune Responses

**DOI:** 10.3390/ijms20184343

**Published:** 2019-09-05

**Authors:** Irina Lyapina, Anna Filippova, Igor Fesenko

**Affiliations:** Department of Functional Genomics and Proteomics of Plants, Shemyakin and Ovchinnikov Institute of Bioorganic Chemistry Russian Academy of Sciences, Moscow 117997, Russia (I.L.) (A.F.)

**Keywords:** cryptic peptides, plant immunity, PAMPs, DAMPs

## Abstract

Plants have evolved a sophisticated innate immune system to cope with a diverse range of phytopathogens and insect herbivores. Plasma-membrane-localized pattern recognition receptors (PRRs), such as receptor-like kinases (RLK), recognize special signals, pathogen- or damage-associated molecular patterns (PAMPs or DAMPs), and trigger immune responses. A growing body of evidence shows that many peptides hidden in both plant and pathogen functional protein sequences belong to the group of such immune signals. However, the origin, evolution, and release mechanisms of peptide sequences from functional and nonfunctional protein precursors, known as cryptic peptides, are largely unknown. Various special proteases, such as metacaspase or subtilisin-like proteases, are involved in the release of such peptides upon activation during defense responses. In this review, we discuss the roles of cryptic peptide sequences hidden in the structure of functional proteins in plant defense and plant-pathogen interactions.

## 1. Introduction

Despite the lack of an adaptive immune system, plants have evolved an innate immune system to cope with the vast majority of potential pathogens. Several models that describe plant response to pathogens have been proposed. The classical “zigzag model” describes two modes of plant resistance mechanisms, PTI (pattern-triggered immunity) and ETI (effector-triggered immunity), both of which lead to the development of systemic acquired resistance (SAR). The innate immunity is based on the recognition of conserved pathogen molecular patterns. These molecules are termed microbe-associated patterns (MAMPs), or also known as pathogen-associated molecular patterns (PAMPs), which are recognized by pattern recognition receptors (PRRs) on the cell surface [1]. Some well-known examples of MAMPs are fragments of flagellin, EF-Tu, DNA, lipoproteins, lipopolysaccharides, and fungal chitin [2]. The recognition of MAMPs and PAMPs by PRRs triggers signaling pathways to downstream defense responses, which is known as a PAMP-triggered immunity system (PTI) [3]. PTI is the first barrier against pathogen invasion, which involves complex physiological changes to provide pathogen resistance. Such changes promote the influx of Ca^2+^, the production of reactive oxygen species (ROS) and NO, the biosynthesis of antimicrobial molecules and defense hormones, the activation of mitogen-activated protein kinases (MAPKs), and the recruitment of transcription factors such as WRKY, BES, ASR, ERF, ORA, or MYC to induce the expression of defense genes [4]. However, co-evolution of plant-pathogen interactions has helped pathogenic microorganisms to acquire special means to avoid receptor recognition and even suppress PTI. To prevent pathogen invasion, an ETI is activated upon recognition of pathogenic effector proteins in the cytoplasm by intracellular nucleotide binding leucine-rich repeat (NB-LRR) proteins. NB-LRR proteins are encoded by R genes [5]. Once activated, ETI can induce the synthesis of signaling molecules, which are then transported from infected to neighboring cells. The perception of these signals leads to an activation of MAP kinases cascades, in a similar way to the PTI mechanism, which in turn triggers the accumulation of molecules such as salicylic acid, as well as transcription of pathogenesis-related (PR)-genes. The released PR-proteins are mostly not pathogen-specific, they may have antibacterial, antifungal, or antiviral activity and are found in the extracellular space as well as in vacuoles [6]. Antimicrobial peptides, such as defensins, cyclotides, thionins, snakins, lipid transfer, and hevein-like proteins, form another substantial part of plant defense mechanisms. These antimicrobial agents are gene-encoded, may consist of 10–50 amino acids and are expressed in various parts of plants [7]. In comparison with PTI, ETI mediates long-lasting responses, such as a hypersensitive response, which ultimately leads to programmed death of the infected cells [8].

The release of endogenous molecules into the extracellular space as a result of damage to host cells can trigger a signal to alert neighboring cells about the danger. The original “zigzag model” does not take into consideration any damage-associated molecular patterns (DAMPs), which indeed play a significant role in the triggering or amplification of host immune responses [9]. Nowadays, endogenous plant signaling peptides have attracted special interest. These peptides are derived from functional and nonfunctional precursor proteins and trigger antiherbivore or antimicrobial defense pathways. Systemin, hydroxyproline-rich systemin, and plant elicitor peptides (PEPs) are the best-studied plant peptide DAMPS [10]. Such plant immune peptides have been recently termed phytocytokines because they have several similarities to metazoan cytokines [11]. These peptides are effective at low concentrations, are perceived by specific receptors, and are mainly derived from inactive protein precursors upon wounding or a pathogen infection. Their precursors are usually small proteins without known functions. For example, AtPEP1 is derived from a 92 amino acid protein precursor [12]. The exact mechanism of Pep1 release was unknown until a recent study by Hander et al., who elaborated on the mechanism involving the calcium-induced activation of metacaspase-4. It has been shown that a conserved AtPEP1 is released from its cytoplasmic precursors PROPEP1 upon wounding by a metacaspase-4 (MC-4), which, in turn, is activated by calcium influx into the damaged cell. Under normal conditions, MC4 is inactive in the cytosol and the protein precursor PROPEP1 is attached to the vacuolar membrane. It is worth considering that MC4-mediated processing of PROPEP1 occurs in leaves, but not in roots [13]. Because *Arabidopsis* has nine metacaspase genes and eight PROPEPs, it may indicate complex protease networks involved in peptide DAMP signaling. Moreover, metacaspases are not only involved in the release of PROPEP family peptides. An extracellular metacapse-9 cleaves out from its precursor a bioactive peptide GRI (GRIM REAPER), which plays a significant role in cell death in *Arabidopsis* [14]. Besides metacaspases, other types of proteases are involved in peptide DAMPs release. A recent study has revealed a novel type of peptide DAMP in cells of *Zea mays*, an immune signaling peptide 1 (Zip1). It is generated by papain-like proteases (PLCPs) after salicylic acid (SA) treatment. Zip1 only induces the transcription of SA-responsive genes [15]. Despite the aforementioned examples, plant proteases involved in the release of plant peptide DAMPs are still yet to be elucidated.

Phytocytokines interact with receptor complexes to downstream their signals. It has been shown that membrane-localized leucine-rich repeat receptor-like kinases (LRR-RLKs) play crucial roles in environmental stress responses, as they are mostly involved in the perception of secreted peptides. For example, in rice, maize, and *Arabidopsis thaliana*, the PEPs transduce their signals by binding to the plant elicitor peptide receptor (PEPR) LRR receptor kinases (RK) — PEPR1 and PEPR2 [12,16]. Both receptors cooperate with the co-receptor BRI1-associated kinase 1 (BAK1), which, in turn, is also involved in developmental regulation through interaction with the plant brassinosteroid receptor BRI [17]. Another well-characterized plant DAMP is an 18 amino acid peptide, systemin, which is recognized by a pair of distinct LRR-RKs—SYR1 and SYR2 [18,19]. This pair of receptors triggers intracellular cascade signaling: depolarization of the plasma membrane, an increase in influx of the Ca^2+^, activation of an MAP kinase and phospholipase A2 activities [20]. Studies in vitro have shown that systemin is released from its precursors by subtilisin-like phytaspases [21]; however, there is no such evidence in vivo.

The precursors of hydroxyproline-rich glycopeptide systemins (HypSys), which represent another family of defense signal peptides, are exposed to hydroxylation of the prolines and glycosylation and are processed through the secretory pathway [22]. This indicates that post-translational modification of immune peptides can play an important role in plant defense response. It is known that some antimicrobial, insecticidal, and quorum sensing peptides belong to the group of ribosomally synthesized and post-translationally modified peptides (RiPPs). Their propeptides undergo post-translational modifications to generate the secreted bioactive RiPP [23]. Nevertheless, the abundance of post-translational modifications in precursors of immune signaling peptides is yet to be elucidated.

Although all the aforementioned examples show the importance of peptide signaling in the plant defense response, the origin and evolution of peptide DAMPs is still unclear. The majority of known peptide DAMPs are generated through the processing of inactive protein precursors. However, modern mass spectrometry and advances in bioinformatics have recently allowed the detection of plant intracellular and extracellular peptide pools, which are generated via degradation pathways of functionally active proteins. A recent study by Filippova et al. showed that a substantial portion (about 10%) of the moss *Physcomitrella patens* secretome peptides contains modifications, for example, hydroxylated proline. The authors of this study also suggest that such peptides might be generated inside the cell and then released via different pathways into the extracellular space or might be cleaved out from their precursors in the extracellular space [24]. However, the role of intracellular and extracellular peptide pools in plant immune response is still poorly understood. Biologically active peptides, which represent fragments of functionally active precursor proteins, are called cryptids [25]. Only three cryptic peptides have been identified in plant tissue [26,27,28]. However, in silico analysis of endogenous peptides has shown that stress conditions lead to a “peptide burst” response, as peptides with antimicrobial potential are formed from functional proteins [29,30]. In the *Arabidopsis thaliana*’s knockout lines of oligopeptidases, PreP and OOP, the accumulation of chloroplast peptides derived from plastid proteins triggers a peptide stress response. This response leads to upregulation of defense-related genes, followed by growth defects and reduced reproductive fitness [31].

The data on plant proteases involved in the formation of peptidomes is still scarce. Most known bioactive peptides are processed by subtilases. For example, RAE2 (regulator of awn elongation), IDA (inflorescence deficient in abscission), RALF 23 (rapid alkalinization factor-23), GLV1 (GOLVEN1), and systemin are all cleaved out from their protein precursors by subtilisin-like proteases [32]. It is also suggested that all of these bioactive peptides are processed in the extracellular space, however, the exact mechanisms of peptides release are yet to be elucidated. The most abundant types of proteases shaping the extracellular pool of endogenous peptides in the moss *Physcomitrella patens* are serine-type (subtilisin-like) proteases, mealloproteases, and aspartic-type proteases. Treatment with salicylic acid leads to an increase in the number of endogenous peptides potentially cleaved by serine-type and aspartic-type proteases [24]. Evidence is emerging about the important role of the plant ubiquitin—proteasome system (UPS) in defense and stress hormone turnover regulation [33,34]. In humans, an aberrant proteasomal activity is observed in cells of patients with systemic lupus erythematosus (SLE), pointing to involvement of the proteasome in disease pathophysiology [35]. Nevertheless, the question of whether endogenous peptides from originally functional proteins can play a substantial role in defense responses as signaling or antimicrobial agents remains to be elucidated. The “dark matter of the proteome” and its formation are nowadays considered one of the main targets in plant peptidomics and proteomics studies.

## 2. Plant Cryptic Immune Peptides

**Figure 1:**
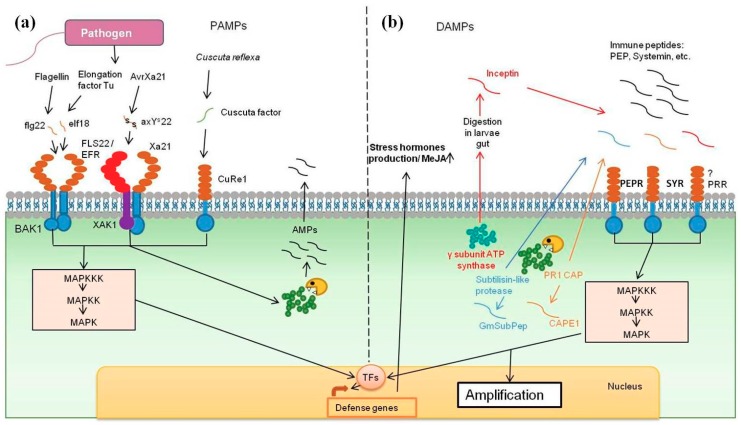
Peptide signals hidden in (**a**) pathogenic and (**b**) host plant proteins.

A growing body of evidence suggests that plant functional proteins contain encrypted signals, which are exposed upon pathogen invasion (Figure 1b). Those encrypted peptides can serve as DAMP molecules. As the methods of mass spectrometry and bioinformatics analyses have improved, several examples of cryptids, which play an important role in the overall fitness of plants, have been identified (Table 1).

### 2.1. Inceptin

The first cryptic peptides discovered in plants were inceptins, which are involved in herbivory defense in *Vigna* and *Phaseoulus* species of legumes. Upon fall armyworm (*Spodoptera frugiperda*) invasion, inceptin (ICDINGVCVDA) is released from the chloroplastic ATP synthase γ-subunit by insect digestive enzymes [28]. Inceptins are 11 to 13 amino acid peptides, each of them containing a core motif of one disulfide-bridged cyclic domain. Asp-3, Asp-20, Cys-8, and the C-terminal Ala are essential amino acids for the bioactivity of inceptins [45]. Eleven-mer and 12-mer inceptins are more bioactive than the 13-mer, however, the 11-mer peptide is the most stable. Upon larvae feeding on the leaves, the peptide is cleaved from the plant сhloroplastic ATP synthase γ-subunit in the midgut of the fall armyworm and with larval oral secretion is transported back onto the plant [28,46]. It is hypothesized that inceptins are products of nonspecific proteolysis, as products of different lengths are produced. The peptide is further recognized as a pathogen elicitor and activates direct and indirect antiherbivore defense responses. Inceptin promotes ethylene production and triggers increases in the defense-related phytohormones, such as salicylic and jasmonic acids [28]. Treatment with inceptin leads to the production of volatile organic compounds, such as indole and methyl salicylate. In turn, the volatiles attract natural enemies of *S. frugiperda*, thus mediating indirect plant defense. Also, inceptins induce expression of protease inhibitors and the production of cinnamic acid, which is an essential substance for phenylpropanoid defense metabolites. The ATP synthase γ-subunit is a conserved enzyme across other plant species. Evidence shows that inceptin peptides are also detected in armyworms larvae oral secretion in maize and tobacco; however, the peptides only trigger defense responses in *Vigna* and *Phaseolus* species [28]. This fact points to a special mechanism of peptide perception in legumes. It is hypothesized that inceptin is recognized through interaction with R protein in cytosol or, otherwise, a PRR can recognize inceptin and transduce the signal [22]. Therefore, as a cryptic peptide, inceptin can be an important regulator of herbivore defense.

### 2.2. CAP-Derived Peptide 1

In tomato plants, a quantitative peptidomic approach was used to identify a defense peptide involved in an antiherbivory response upon wounding. An 11 amino acid cryptic peptide was derived from the C-terminal end of the tomato PR1b preproprotein of the pathogenesis-related 1 proteins (CAP) superfamily and named CAP-derived peptide 1 (CAPE1) [26]. A marker gene for the salicylic acid signaling pathway was processed to release CAPE1 peptide upon wounding or methyl jasmonate (MeJA) treatment. Pretreatment of tomato leaves with synthetic CAPE1 caused a suppressed larval growth of *Spodoptera litura* and increased resistance to the bacterial pathogen *Pseudomonas syringae* pv tomato DC3000 compared with the control samples. It also led to a burst of H_2_O_2_ production. The treatment of a cell with CAPE1 alters the transcriptional profile of a cell, as gene expression analysis has shown that it induces the expression of known genes of antiherbivory response, bacterial defense, and systematic acquired resistance. As a result, an increased level of jasmonic acid (JA) and SA hormones was observed [26]. Thirty novel putative CAPE1-like peptides with a conserved proline-rich PxGNxxxxxPY motif were identified in *Viticeae*, *Solanoideae*, *Fabaceae*, *Brassicaceae*, and *Nicotianoideae*. This means that CAPE1 and its homologs represent a conserved peptide family that is involved in plant defense response across various plant species. Moreover, in *Arabidopsis*, it has been recently discovered that AtCAPE, which is a homolog of CAPE1 in tomato plants, is involved in the regulation of salt stress responses [47]. CAPE1 is considered a DAMP molecule that induces an immune response similar to other bioactive peptides, such as systemin, RALF, PEPs, and HypSys [26].

### 2.3. Glycine Max Subtilase Peptide

Recently, an endogenous cryptic peptide that originated from extracellular subtilisin-like protease has been isolated in soybean and named *Glycine max* Subtilase Peptide (GmSubPep) [27]. The cryptic sequence is located within the protease-associated (PA) domain located within the S8 peptidase region of the gene Clyma18g48580, which is unique for legumes. GmSubPep consists of 12 amino acids. The amino acids at positions 10 (arginine) and 12 (histidine) are essential for the bioactivity of this peptide, whereas the most represented amino acids are proline and arginine [27]. The authors suggest that subtilase is activated upon pathogen attack and GmSubPep is further released to the apoplast to downstream signals for the immune response. Under biotic stress, GmSubPep enhances the expression of genes involved in the defense response in plant cells [27]. The treatment with synthetic GmSubPep resulted in induced expression of defense genes, such as Cyo93A1 (involved in phytoalexin synthesis), a pathogenesis-related gene, chitinase 1b-1 (Chib-1b) and PDR12 (a salicylic-acid-inducible ATP-binding cassette transporter), and chalcone synthase (achs) (involved in phytoalexin production). The peptide fraction was able to change the pH of the soybean suspension throughout the course of the experimental procedure. Treatment with a synthetic analogue showed that the pH of the suspension altered within 10 min, reaching the maximum alkalizing response in 15 min. Therefore, the peptide is bioactive at an extremely low concentration. This was in concordance with the physicochemical properties of other plant peptide elicitors, such as AtPEP and ZmPEP, which are active at nanomolar concentration. Also, because the treatment of tomato plants, *Arabidopsis*, and tobacco with GmSubPep did not show any alkalinization affect, the authors suggested that GmSubPep was unique only for soybean as a systemin for tomato plants [48].

The examples of known cryptic peptides show that proteins can have dual functions and be an additional source of secondary DAMPs in case of pathogen attack. There is also evidence that high mobility group box 1 (HMGB1)-like proteins, which are highly conservative, found in all eukaryotes, and are involved in chromatin architecture regulation, can be found both in the nucleus and in the cytosol [49,50,51]. It has been shown that AtHMGB3, which is homologous to metozoan HMGB1, is released to the apoplast and acts like a DAMP molecule and triggers host immune activation during infection by necrotrophs or herbivores. The downstream signals are similar to those triggered by AtPep1 [10]. Therefore, HMGB-proteins might switch their initial function and act like primary DAMPs molecules. It is evident that the whole extent of protein’s functions and signals encrypted in them is yet to be discovered.

## 3. Peptide Signals Hidden in Phytopathogenic Proteins

One of the most important aspects of the classical zigzag model of plant defense response is the perception of MAMPs and PAMPs by different PRRs located on the cell surfaces (Figure 1a). The range of substances capable of inducing such signaling is quite broad, but of special interest are endogenous peptides that originate from functional pathogen proteins and are capable of triggering immune responses (Table 1).

### 3.1. Flagellin-Derived Peptides

One of the well-known examples of PAMPs is a peptide derived from the N-terminus region of the conserved microbial protein flagellin. Flagellin is present in many bacteria species and is pathogenic for both plants and animals. Plants have developed a system enabling the recognition of flagellin fragments by host PRRs. In Arabidopsis, the flg22 peptide is recognized by the leucin-rich repeat receptor kinase FLAGELLIN SENSING2 (FLS22) [4]. Such PRRs were also found in tomatoes, tobacco, and rice [52,53,54]. Felix and co-workers were the first to demonstrate that the conserved sequences of 15–22 amino acids at the N-terminus play a role as elicitor, causing a specific response in plants [37]. To prove that the given peptide provokes the plant immune response, mutant lines were generated, which produced peptides with replaced amino acids. These mutants appeared to have less pathogenic activity as well as reduced mobility, suggesting the essential importance of the sequence not only for MAMP activity, but also for the function of the protein itself [55]. Another peptide encrypted in a distinct part of bacterial flagellin and its corresponding receptors, flgII-28 and FLS3, respectively, were recently discovered in tomato plants [56]. A basal land plant—the moss *Physcomitrella patens*—is insensitive to the elf18 peptides, flg22 peptides, and full-length flagellin proteins, and it lacks EFR and FLS2 receptors [1,42]. However, Bressendorf et al. showed that a recognition system was activated in response to other known PAMPs, such as chitin and bacterial peptidyl glycan [42]. This fact raises the question of whether the conservativity of different PAMPs evolved through years of plant-pathogen interactions. We suggest that vascular plants have acquired a more sophisticated immune system in comparison with nonseed plants, which are inhabited in a less stressful environment.

Apoplastic glycosidases, specifically ß-galactosidases 1 (BGAL1), hydrolyze glycans carried on the flg22 peptide site of the protein precursor, flagellin. Deglycosylation makes flagellin assessable for host apoplastic proteases, which further cleave out the peptide. However, specific classes of these proteases still remain to be disclosed. Therefore, plant extracellular proteases not only play an important role in the generation of native bioactive peptides but also act as an intermediate between pathogens elicitors and host PRRs [57].

### 3.2. Elf18

Similar to flagellin and flg22, elf18 is an 18 amino acid peptide cleaved from the N-terminus of another abundant and common bacterial protein, provoking an immune response in *Arabidopsis thaliana* and other plants of this family [38]. Using *Arabidopsis thaliana* as a model plant, it was shown that the microbial elongation factor Tu releases elf18, which is recognized by plant leucine-rich repeat receptor-like kinase EF-Tu receptor (EFR) [39]. Arabidopsis mutants lacking EFR expression were more easily transformed by *Agrobacterium*, indicating the important role of this receptor in plant defense. Interestingly, the transient expression of the EFR gene into the plant of another family, *Nicotiana benthamiana*, lacking this PRR, resulted in a specific response to treatment with the elf18 [39].

In rice, the elf18 peptide did not activate any response, in contrast to another peptide, termed EFa50, derived from the middle region of EF-Tu, which induced H_2_O_2_ generation and callose deposition. Also, a conservative peptide, flg22, from a virulent bacterial strain, was not recognized by rice PRRs, in contrast to purified flagellin fragments from an avirulent strain, as well as a flagellin-deficient derivative of that strain, which induced the expression of several immune-related genes, including PAL, Cht-1, and PBZ1 [58]. Both of the abovementioned receptor-like kinases formed a complex with BAK1—BRI1-associated receptor kinase 1—to initiate immune response cascades in plant cells [59,60].

### 3.3. Sulfated Peptide RaxX

A substantial group of antimicrobial and insecticidal peptides belong to the class of ribosomally synthesized and post-translationally modified peptides (RiPPs). This group also includes sulfated derivatives of the microbial protein AvrXa21, which induces the XA21-mediated immunity X (RaxX) in rice. The transmembrane immune receptor-like kinase XA21, which shares homology to Toll-like receptors and flg22 and elf18 receptors, was the first PRR predicted in rice in 1995 [40]. It was only later found that the sulfated peptide from *Xanthomonas oryzae* protein appears to be a ligand to this receptor [61]. A 16 amino acid peptide, RaxX, is derived from its precursor protein. However, the interaction with the Xa21 immune receptor is intensified by tyrosine sulfation. The biosynthesis of this peptide was previously unknown until a recent study by Luu et al., who showed that the precursor peptide (proRaxX) is cleaved at the Gly–Gly site, processed, and secreted by the protease/transporter component B (PctB). RaxX mimics plant peptide hormones, PSY1, and PSY1-like proteins, as it shares amino acid sequence homology and exogenous application of the sulfated RaxX16 promotes root growth. However, the synthetic PSY1 peptides derived from *Arabidopsis* and rice do not induce an XA21-dependent immune response [62].

### 3.4. Pep-13

In parsley, a 13 amino acid peptide, Pep-13, derived from *Phytophthora sojae* glycoprotein GP42, triggers an immune response [41]. Mutation analysis showed that the peptide elicitor is produced from the same region of the protein precursor, in which the domain essential for transglutaminase (TGase) activity is located. Although the GP42 protein shares biochemical characteristics with known mammalian TGases, it has no sequence homology to any of them but is highly conserved among oomycete *Phytophthora* TGases. Moreover, treatment with synthetic Pep-13 activated the synthesis of phytoalexins in potato plants [63].

### 3.5. Cuscuta Factor

Parasitic plants could also be a source of PAMPs. Recent research has shown that the exceptional resistance of tomato plants to *Cuscuta reflexa* is mediated by specific recognition of some “Cuscuta factor” (CuF), which then appears to be a cell-wall associated peptide, by a cell surface receptor-like kinase CuRe1 in a similar way to the PAMP/PRR system, developing during microbial attack. This 2 kDa peptide induces the production of ROS and the biosynthesis of the stress-related phytohormone ethylene. Transient expression of the gene coding the PRR CuRe1 in tobacco also results in resistance of the previously insensitive host plant to *Cuscuta reflexa*, suggesting the existence of the same downstream signaling cascades as in the case of plant/PAMP interaction [64,65].

### 3.6. Csp22, Csp15

Bacterial cold shock proteins possess a highly conserved RNP-1 motif, which is also present in eukaryotic RNA- and DNA-binding proteins. A native 22 amino acid peptide, csp22, as well as a synthetic 15 amino acid peptide, csp15, derived from the conserved domain bacterial cold shock proteins induce a specific response in tobacco [43]. However, the mechanisms of their perception have not yet been discovered.

### 3.7. RxLR Avr Effectors

Plant pathogens RxLR Avr effectors, which activate ETI, are highly variable except several conserved motifs at the C-terminus of their sequences. In *Solanum tuberosum*, most of these domains, named W, Y, T, of RxLR effectors, including Arv1 of *Phytophthora infestans,* are required for the effector activity and are also recognized by plant resistance (R) proteins [44].

## 4. Functional Proteins as a Source of Antimicrobial Peptides

**Figure 2:**
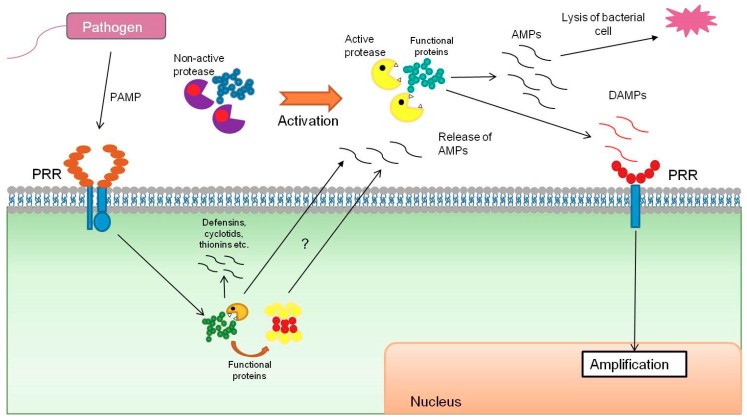
Antimicrobial peptides from functional proteins.

Antimicrobial peptides (AMPs) represent one of the main plant protective barriers against attack by phytopathogens in different phylogenetic groups. AMPs have been isolated from various organs, such as the root, seeds, inflorescences, stem, and leaves [66]. Plant species can comprise hundreds of different AMPs, deriving from special preproteins and secreted via the endoplasmic reticulum for further maturation. Most of the known plant AMPs belong to the group of amphiphilic cysteine-rich peptides, the size of which does not exceed 10 kD. The most-studied and main families of AMPs are defensins, cyclotids, thionins, lipid transfer proteins, and hevein-like proteins. They can be either constitutively expressed and stored or their expression can be induced under biotic and abiotic stress conditions [67]. However, recent advances in the field of proteomics and peptidomics point to surprising evidence that functional proteins can be an additional source of antimicrobial peptides (Figure 2) [29,68,69].

In silico analysis of the amino acid sequences of proteins allows the prediction of potential antimicrobial peptides, which are fragments of long polypeptide protein chains [70]. Several bioinformatic programs are used for the prediction of antimicrobial activity: Kamal, CAMP, and iAMPpred. These algorithms analyze amino acid sequences within the proteins and, according to the physicochemical properties of other well-studied antimicrobial peptides, search for protein fragments with potential antimicrobial activity [70].

In animal cells, a great example of such an encrypted peptide is oligoventin, which was processed by proteolytic proteasomal degradation of a functional Nedd4 E3 precursor protein. This cryptic peptide acts as a host defense peptide (HDPSs) in arachnids [71]. Also, frog-skin-derived antimicrobial peptides are released from conservative preproprotein as a result of specific prohormone signaling [72]. Plant functional proteins can also be an additional source of antimicrobial peptides. For example, in *Glycine max* cells, enzymatic proteolysis of D-myo-Inositol-3-phosphate synthase leads to the generation of an antimicrobial peptide, which mediates the resistance to Asian rust spores (*Phakopsora pachyrhizi*). The transformation of *Nicotiana tabacum*, *Solanum tuberosum*, and *Brassica rapa* cells by frog-skin-derived antimicrobial peptides, esculentin-1, dermaseptin SI, and hCAP18/LL37, enables the acquisition of resistance to pathogenic fungi and bacteria [70].

Previously, using mass spectrometry analysis of gametophore, protonema, and protoplast cells, we detected 20,000 unique endogenous peptides from functional proteins [30]. In silico analysis showed that 117 peptides had potential antimicrobial activity. The antimicrobial activity of five synthesized peptides, fragments of functional proteins, has been experimentally validated against the gram-positive bacteria *Bacillus subtilis* SHgw and *Clavibacter michiganensis* pv. *michiganensis* and gram-negative bacteria *Escherichia coli* K12 and *Xanthomonas arboricola* 3004. Three of the peptides, unique to protoplasts, represented fragments of ribosomal proteins and had the highest predicted antimicrobial potential. They inhibited the growth of both gram-positive and gram-negative bacteria. The peptide unique to protonemata, which is derived from the “predicted” protein A9RR37, inhibited the growth of the gram-negative *X. arboricola* 3004 and the gram-positive *C. m.* pv. *michiganensis*. Another peptide, which was a fragment of the component Q6YXMS of photosystem II, exhibited inhibitory activity against the gram-negative bacterium *E. coli* K12 [68]. We suggest that these AMPs are generated from functional proteins upon stress conditions and contribute to the plant defense response as an additional barrier against pathogens. We recently performed another search for encrypted antimicrobial peptides within protein sequences [29]. The composition of extracellular peptidome was dramatically altered under stress hormone treatment (MeJA). Stress conditions led to the generation of cryptic peptides, which had antimicrobial activity and were products of proteolysis of functionally active proteins. Using a serial dilution method with *E. coli* and *B. subtilis* bacteria, it was revealed that the one-hour MeJA-treated secretomes restricted bacterial growth. The observed bacteriostatic effect indicated that MeJA had a signaling role rather than functioning as an antimicrobial compound. The addition of the protease inhibitor cocktail to prevent proteolytic degradation of secreted proteins led to a significant decrease of the bacteriostatic effect. The subsequent bioinformatic analysis of peptides identified in the cell and secretome of *P. patens* revealed that 3.5% of all secretome peptides had potential antimicrobial properties. These peptides originated from functional protein precursors involved in different processes. Four percent of peptides identified in the cell peptidome were also predicted as potentially antimicrobial peptides. Treatment with the stress hormone methyl jasmonate increased the number of potential antimicrobial peptides, pointing to a potential role in plant stress response. The biological activity of eight of the predicted AMPs was experimentally verified using a serial dilution method with *E. coli* and *B. subtilis* bacteria. The minimum inhibitory concentration (MIC) for the two peptides was comparatively low, 64 and 16 µg/mL (INIINAPLQGFKIA), respectively, compared with the positive control, the well-known antimicrobial standard melittin (8 µg/mL) [29]. This further supports the evidence that cryptic peptides act as quick-release antimicrobial agents from functional proteins under stress conditions in plants. We hypothesize that plants have evolved such a quick immune response system to provide an extra tool to cope with stress conditions. Further studies of antimicrobial peptides, which are encrypted within functional protein fragments and generated by proteolytic degradation, are needed to understand genome plasticity and principles of biodiversity.

## 5. Perspectives

Peptide-receptor recognition is a complex process, as peptides can be recognized by more than one receptor or, vice versa, one receptor can recognize more than one signaling peptide. For instance, CLV3 involved in processes of plant growth and development can be recognized by various complexes: the homomer CLV1-CLV1, the heteromer CLV2-CRN (CORYNE), and the multimer CLV1-CLV2-CRN receptors (Som) and RECEPTOR-LIKE PROTEIN KINASE 2 (RPK2) and BARELY ANY MERISTEM 1 and 2 (BAM 1 and 2) [73]. Therefore, the number of known peptide-receptor ligands is still not vast. Peptidomics allows a comprehensive analysis of endogenous peptides to be performed, and, unlike its ancestor, proteomics, it represents a relatively new area of research. Recent studies provide new insight into the role of endogenous peptides that originated from functional proteins in plant defense. Although some of the thousands of endogenous peptides found in plant cell and tissues are simply intermediate products in protein degradation, others have distinct biological functions inside or outside the cell. Moreover, the accumulation of endogenous peptides caused by the knockout of oligopeptidases, which are responsible for further peptide degradation, leads to the emergence of a specific defense phenotype, which suggests that the products of protein breakdown are not just passive but contain important biologically active elements. Future studies will help to establish the fundamental role of cryptic peptide sequences in plant defense and other aspects of plant biology.

## Figures and Tables

**Table 1:** Cryptic immune signals.

Name	Precursor	Receptor	Reference	Peptide Sequence
Plant cryptic peptides				
Inceptin	ATP synthase γ-subunit	Inceptin receptor (INR)	[28,36]	ICDINGVCVDA
CAP-derived peptide 1 (CAPE1)	PR1b preproprotein of pathogenesis-related 1 proteins (CAP) superfamily	?	[26]	PVGNWIGQRPY
Glycine max Subtilase Peptide (GmSubPep)	Subtilisin-like protease	?	[27]	NYYDKHQLTRGH
PAMPs				
flg22	Flagellin	FLAGELLIN SENSING 2 (FLS22)	[4,37]	QRLSTGSRINSAKDDAAGLQIA
elf18	Elongation factor Tu	EF-Tu Receptor (EFR)	[38,39]	SKEKFERTKPHVNVGTIG
RaxX	preRaxX	XA21	[11,40]	DYPPPGANPKHDPPPR
Pep-13	Glycoprotein GP42	R protein	[41]	VWNQPVRGFKVYE
Cuscuta factor (CuF)	?	Cuscuta receptor 1 (CuRe1)	[42]	?
Csp22, csp15	Cold shock protein	?	[43]	(AVGT)VKWFNAEKGFGFITP(DDG)
RxLR Avr effector	Avr1 etc.	R protein	[44]	?

## Abbreviations

PRR	Pattern recognition receptor
RLK	Receptor-like kinases
PAMP	Pathogen-associated molecular pattern
DAMP	Damage-associated molecular pattern
PTI	Pattern-triggered immunity
ETI	Effector-triggered immunity
NB-LRR	Nucleotide binding leucine-rich repeat
PEP	Plant elicitor peptide
LRR-RLK	Leucine-rich repeat receptor-like kinases
PEPR	Plant elicitor peptide receptor
BAK1	BRI1-associated kinase 1
SA	Salicylic acid
MeJA	Methyl jasmonate
AMP	Antimicrobial peptides
